# Mixed squamous cell and glandular papilloma of the lung―A case report and literature review in Japan

**DOI:** 10.1016/j.ijscr.2020.02.021

**Published:** 2020-02-13

**Authors:** Yoshihito Iijima, Yuki Nakajima, Hiroyasu Kinoshita, Hirohiko Akiyama, Yu Nishimura, Tomomi Hirata

**Affiliations:** Division of Thoracic Surgery, Saitama Cancer Center, Saitama, Japan

**Keywords:** Mixed squamous cell and glandular papilloma, Lung neoplasm, Segmentectomy, Case report

## Abstract

**Introduction:**

Mixed squamous cell and glandular papilloma (MSGP) of the lung is rare neoplasm, only 19 cases previously reported in Japan. MSGP is one of three separate categories of solitary endobronchial papillomas, which account for less than 0.5 % of all lung tumors. MSGP is rarest of the three. We present a rare case of MSGP and review the literature in Japan.

**Presentation of case:**

A 49-year-old man presented with a nodulous shadow in the right lower lung field identified on a chest X-ray performed for back pain. Computed tomography demonstrated a well-circumscribed, 9-mm mass in the S8 segment of the right lower lung lobe. Positron emission tomography showed accumulation of 18F-fluorodeoxyglucose in the tumor with a standardized uptake value of 2.29. Following thoracoscopic partial resection, adenocarcinoma was diagnosed by frozen section, and radical segmentectomy of S8 was performed. The final diagnosis was mixed squamous cell and glandular papilloma. The patient had an uncomplicated postoperative course and remains asymptomatic 3 years after the procedure.

**Discussion:**

The etiology and pathological characteristics of MSGP remain unclear. There has been no report of recurrence in MSGP cases treated with complete resection; thus, limited resection that preserves pulmonary function is desirable. Further data including new cases are required.

**Conclusion:**

We present a rare case of mixed squamous cell and glandular papilloma (MSGP) of the lung and review the literature in Japan. Limited resection that preserves pulmonary function, while ensuring complete resection, is desirable. Further investigations with new cases are required.

## Highlights

• Mixed squamous cell and glandular papilloma (MSGP) of the lung is an extremely rare entity.
• Preoperative diagnosis and intraoperative diagnosis by frozen section of MSGP are very difficult.
• Complete resection and limited resection should be considered for MSGP.

## 1 Introduction

Mixed squamous cell and glandular papilloma (MSGP) is an endobronchial papillary tumor showing a mixture of squamous and glandular epithelium, with at least one-third of the tumor compring the glandular component [1]. MSGP is one of three separate categories of solitary endobronchial papillomas (SEPs): (i) squamous cell papilloma, (ii) glandular papilloma, and (iii) MSGP (mixed papilloma), which Flieder described in 1998 [2]. SEPs account for less than 0.5 % of all lung tumors [2,3] and approximately 7% of all benign epithelial and mesenchymal lung tumors [3]. MSGP accounts for 15.6–20.4% of SEPs [4,5]. MSGP is an extremely rare neoplasm; only 19 cases prior to the current case have been reported in Japan. Thus, its clinicopathological features remain unclear. Herein, we present a rare case of MSGP and review the literature in Japan.

The work has been reported in line with the SCARE criteria [6].

## 2 Presentation of case

**Fig. 1:**
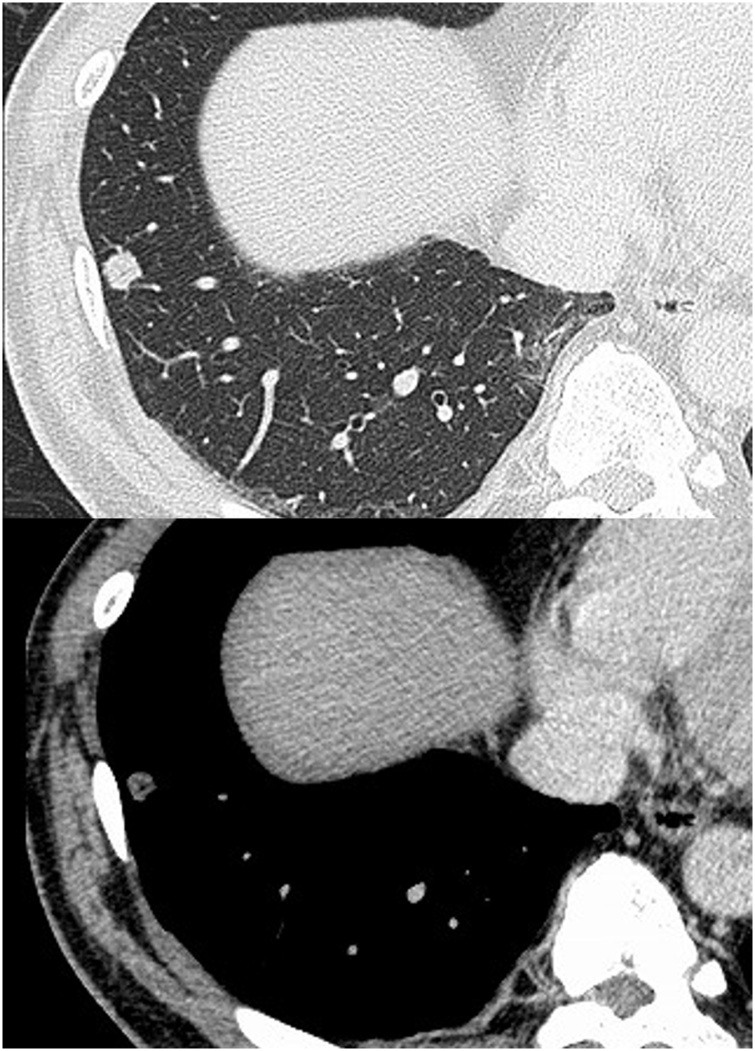
**Radiological findings.** Computed tomography showed a well-circumscribed, 9-mm mass in the S8 segment of the right lower lung lobe.

**Fig. 2:**
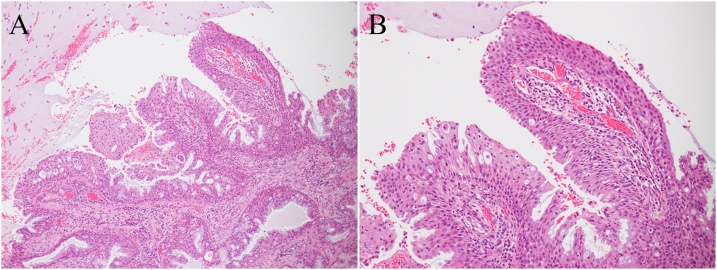
**Microscopic findings.** (A) Intermixed and proliferating ciliated epithelium, goblet cells, and squamous epithelium were present along with alveolar epithelial replaced by papillary structures. Hematoxylin and eosin (HE) staining, magnification ×10. (B) HE staining, magnification ×20.

A 49-year-old man presented to our division for evaluation of a nodulous shadow in the right lower lung field on chest X-ray performed for back pain. He had a smoking history of 45 pack-years. Chest computed tomography (CT) showed a well-circumscribed, 9 mm mass in the S8 segment of the right lower lung lobe (Fig. 1). Positron emission tomography (PET)/CT imaging showed 18F-fluorodeoxy glucose (FDG) uptake of the tumor with a standardized uptake value of 2.29. Serum tumor markers, including carcinoembryonic antigen, squamous cell carcinoma antigen, cytokeratin 19 fragment, and pro-gastrin-releasing peptide were within the normal ranges. The patient strongly desired a definitive diagnosis and thus underwent thoracoscopic partial resection. The nodule was white, measured 11 × 8 mm, and showed well-defined borders. Frozen section biopsy results led to a diagnosis of adenocarcinoma. No metastasis in the hilar lymph node (#11) was identified, and radical segmentectomy of S8 was performed. Histopathologic examination showed intermixed and proliferating ciliated epithelium, goblet cells, and squamous epithelium along with alveolar epithelial replacement by papillary structures (Fig. 2). Weak cell atypia and low proliferative ability were observed in the cells. The tumor cell nuclei were relatively homogeneous, and mitoses were rare. Immunohistochemical staining showed p40 positivity in the areas of squamous epithelial differentiation. Ki-67 was positive on the basal side of the areas of squamous epithelial differentiation. The tumor cells were negative for thyroid transcription factor-1. The final diagnosis was mixed squamous cell and glandular papilloma. The patient had an uncomplicated postoperative course and remains asymptomatic 3 years after the procedure.

## 3 Discussion

MSGP of the lung is extremely rare; thus, its etiology and clinicopathological characteristics remain unclear. Only 20 cases have been reported in Japan, including the present case (Table 1) [5,[7], [8], [9], [10], [11], [12], [13], [14], [15], [16], [17]]. The patients’ ages ranged from 34 to 84 years (mean, 60.7 years). Ten lesions were located in the right lung and 10 in the left lung. Eight lesions were centrally located and 12 were peripheral.

**Table 1:** Mixed squamous cell and grandular papilloma reported in Japan.

No.	Age	Sex	Smoking	Site	Location	Size (mm)	Tumor marker elevation		SUV max	Bronchoscopic cytology	TBLB/TBB/CTNB	Frozen Section	Therapy	LND	HPV	Reference
							CEA	SCC								
1	63	F	+	RUL	C	n/a	n/a	n/a	n/a	n/a	Papilloma	n/a	Lob	−	n/a	7)
2	66	F	−	RML	C	30	n/a	n/a	10.6	n/a	atypical squamous cells	Papilloma	Lob	−	n/a	8)
3	84	F	−	RUL	C	10	−	−	n/a	n/a	MSGP	−	Endoscopic excision	−	No (IHC)	9)
4	68	F	+	LUL	P	10	n/a	n/a	No significant increase	−	−	Ad	Wed	−	No (ISH, PCR)	10)
5	55	F	−	LUL	P	26	n/a	n/a	9.01	no tumor cells	n/a	n/a	n/a(VATS)	n/a	n/a	11)
6	74	F	−	Rt-S5	P	20	+	+	n/a	n/a	mucoepidernoid carcinoma	mucoepidermoid carcinoma	Lob	1b	No (ISH)	12)
7	72	F	−	Rt-S6	P	20	−	+	5.8	n/a	no malignancy	SCC	Lob	2a	n/a	
8	40	F	−	Lt-S10	P	30	−	+	11.2	class Ⅲ	n/a	Papilloma	Lob	2a	No (ISH)	
9	74	F	−	Rt-S8	P	10	n/a	n/a	n/a	−	−	no malignancy	Wed	−	No (IHC)	12)
10	70	F	−	Lt-S10	P	27	+	n/a	n/a	no tumor cells	no tumor cells	Papilloma	Wed	−	n/a	12)
11	59	M	+	Lt-S8	C	20	−	−	n/a	atypical squamous cells	n/a	Papilloma	Seg	−	n/a	12)
12	60	M	+	Rt-S5	C	18	+	n/a	3.4	class Ⅳ	n/a	Papilloma	Lob	−	n/a	14)
13	49	M	+	Lt-S6	C	30	−	−	n/a	atypical squamous cells	n/a	n/a	Lob	n/a	No (ISH, PCR)	5)
14	41	M	−	Lt-S6	C	30	n/a	+	n/a	n/a	MSGP	−	Lob	2a?	n/a	15)
15	72	M	+	Lt-S6	C	n/a	−	+	n/a	n/a	SCC	n/a	Seg	2a	No (IHC)	12)
16	49	M	+	Rt-S8	P	30	+	n/a	14.8	SCC suspected	SCC	n/a	Lob	2a	No (IHC)	
17	34	M	n/a	Lt-S10	P	15	n/a	n/a	n/a	class Ⅲ	mucoepidernoid carcinoma	AdSq	Lob	2a	n/a	12)
18	70	M	−	Rt-S9	P	28	+	+	6.09	class Ⅱ	no malignancy	n/a	Lob	2a	n/a	16)
19	64	M	+	Lt-S8	P	30	+	+	15.17	n/a	−	AdSq	Lob	+	n/a	17)
20	49	M	+	Rt-S8	P	9	−	−	2.29	−	−	Ad	Seg	1b	No (IHC)	Present Case

M: male, F: female, RUL: right upper lobe, LUL: left upper lobe, C: central, P: peripheral, n/a: not available, TBLB: trans-bronchial lung biopsy, TBB: trans-bronchial biopsy, CTNB: computed tomography guided nodule biopsy, n/a: not available, MSGP: mixed squamous cell and glandular papilloma, SCC: squamous cell carcinoma, Ad: adenocarcinoma, AdSq: adenosquamous carcinoma, Lob: lobectomy, Seg: segmentectomy, Wed: wedge resection, VATS: video-assisted thoracic surgery, LND: lymphonode dissection, IHC: immunohistochemistry, ISH: in situ hybridization, PCR: polymerase chain reaction.

Inamura et al. reported that MSGP is seen predominantly in males, and that a history of smoking and human papilloma virus (HPV)-negative status may indicate an etiological association between MSGP and tobacco smoke [5]. Furthermore, they note that there are many non-smoking patients with peripheral type MSGP and that central and peripheral tumors may have different etiologies. However, unlike in previous reports, there was no gender difference and no difference in smoking history identified in our literature review. A history of smoking was more frequent among male than female patients, and non-smokers were more likely to have peripheral tumors. HPV DNA has been detected in many cases of squamous epithelial papilloma, but there are no reported cases of HPV DNA identified in MSGP. In our literature review, eight of 20 patients tried to identify HPV DNA, but none of them were confirmed to be HPV positive. In the present case, postoperative examinations such as HPV in situ hybridization and indirect immunohistochemical staining of p16 to suggest the absence of HPV were recommended after surgery for patient but the patient refused.

It is difficult to diagnose MSGP and exclude malignancy preoperatively. Only three patients, all of whom had central tumors, reported receiving a definite diagnosis of papilloma before surgery. Four cases were diagnosed as lung cancer before surgery (two squamous cell carcinomas, two mucoepidermoid carcinomas) [12]. Peripheral MSGP is more difficult to diagnose compared to central MSGP because the tumor often cannot be directly observed with a bronchoscope and sufficient biopsy samples cannot be easily obtained. Kadota et al. stated that it is difficult to diagnose MSGP by cytology alone because there is the possibility of contamination of the cytological specimens from normal bronchial epithelial cells or squamous metaplastic cells outside of the main lesion [13]. Intraoperative frozen section was performed in 12 cases; five were diagnosed as papilloma, but six were diagnosed as carcinoma, which shows that accurate diagnosis is challenging. In addition to the difficulty of preoperative pathological diagnosis, the absence of specific imaging findings and lack of consistent tumor markers and FDG accumulation findings make it more difficult to exclude malignancy.

Concerning treatment, lung resection was performed in 19 cases; in the remaining case, bronchoscopic resection was performed. Lobectomy was performed in 12 cases. Since MSGP is classified as a benign tumor and there is no report of recurrence in the case of complete resection. If a definitive diagnosis is reached before surgery, limited resection that preserves pulmonary function is desirable. However, as described earlier, it is difficult to achieve preoperative diagnosis and there is also a risk of preoperatively misdiagnosed malignancy [2,18,19]. Thus, a surgical procedure designed for lung cancer is most often chosen. In this case, we thought that the possibility of malignant tumor was low based on preoperative imaging findings. However, the tumor was diagnosed as adenocarcinoma by intraoperative frozen section, and radical segmentectomy was performed. In contrast, there are reports of cases where malignant findings were not observed on preoperative cytological and histological samples but exclusion of malignancy was difficult based on imaging findings, leading to the performance of radical resection [12,16]. Further data including additional new cases are required. The present patient showed no evidence of recurrence 3 years after surgery, but meticulous follow-up examinations will be continued.

## 4 Conclusion

We report an extremely rare case of MSGP in the lung. The etiology and pathological characteristics of MSGP remain unclear. There has been no report of recurrence in cases treated with complete resection, thus limited resection that preserves pulmonary function is desirable. Further investigations with additional new cases are required.

## Funding

All authors have no funding of research.

## Ethical approval

This study was approved by the institutional review board in June 2019 (approval number: 950), and the need to obtain informed consent was waived.

## Consent

Written informed consent was obtained from the patients for publication of this case report and accompanying images.

## Author contribution

Yoshihito Iijimaa carried out the operation, wrote this manuscript and carried out data collection. Yuki nakajima, Hiroyasu Kinoshita, Hirohiko Akiyama, Yu Nishimura and Tomomi Hirata carried out the revision of the manuscript.

## Registration of research studies

N/A.

## Guarantor

Yoshihito Iijima.

## Provenance and peer review

Not commissioned, externally peer-reviewed.

## Declaration of Competing Interest

All authors report no conflict of interest.
